# Neural Correlates of Explicit Outcome Expectation Effects: An Activation Likelihood Estimation Meta‐Analysis

**DOI:** 10.1002/hbm.70594

**Published:** 2026-07-02

**Authors:** Junnan Zhou, Yiyang Xu, Tieyan Li, Xingyue Yang

**Affiliations:** ^1^ College of Acupuncture‐Moxibustion and Tuina Beijing University of Chinese Medicine Beijing China; ^2^ Graduate School Beijing University of Chinese Medicine Beijing China

**Keywords:** activation likelihood estimation, brain imaging, explicit outcome expectation effects, meta‐analysis

## Abstract

To investigate the characteristics of brain region activation associated with explicit outcome expectation effects in treatment contexts, and to identify core brain regions consistently involved across diverse experimental paradigms. A systematic search was conducted for studies published from January 1, 2014, to December 31, 2025. Functional magnetic resonance imaging (fMRI) and positron emission tomography (PET) studies investigating treatment expectation effects were included. In neuroimaging paradigms, these effects are commonly induced through explicit outcome expectations via verbal instructions or contextual cues. Risk of bias was assessed using the ROB 2.0 and ROBINS‐I tools. Activation likelihood estimation (ALE) meta‐analysis was performed using GingerALE 3.0.2 based on whole‐brain activation coordinates. Results were visualized using Mango software. Sensitivity analyses were performed by excluding the study with the highest risk of bias and using a leave‐one‐study‐out approach. Additionally, a qualitative synthesis of activation foci was conducted across four domains to address paradigm heterogeneity. A total of 21 articles with 855 subjects were included in this study, consisting of 17 randomized controlled trials and 4 non‐randomized studies. The risk of bias assessment indicated that only five RCTs were rated as low risk, while approximately three‐quarters of studies showed some concerns or high risk. Most studies modulated expectations through interventions such as verbal instruction and pain stimulation. Convergent neuroimaging studies implicated the insula, frontal lobe, striatum, and additional relevant brain regions. The ALE meta‐analysis identified two significant activation clusters located in the caudate nucleus/lentiform nucleus and the red nucleus. Sensitivity analyses indicated that the majority of findings remained stable. Qualitative domain‐based synthesis further revealed consistent striatal activation across all domains, together with distinct domain‐specific activation patterns. The findings of this study suggest that explicit outcome expectation effects, as a core operationalized component of treatment expectation, do not rely on a single brain region but emerge from distributed and dynamically interacting neural systems that depend on reward expectation and information integration. The coordinated activity of multiple brain regions collectively supports the generation of explicit outcome expectations. The striatum serves as a shared core substrate across different expectancy domains, while domain‐specific activation reflects functional specialization for distinct contexts. These findings provide important evidence for elucidating the neural correlates of explicit outcome expectation effects and establish a foundation for further brain mechanism‐based intervention strategies.

**Trial Registration:** PROSPERO: CRD420251074894

## Key Points

- Using an integrative ALE approach, this study provided a systematic and consistent characterization of brain activation patterns associated with explicit outcome expectations in treatment contexts.
- This meta‐analysis identified the caudate nucleus/lentiform nucleus as a core neural substrate with the red nucleus emerging as an additional significant cluster.

## Graphical abstract

This ALE meta‐analysis of brain imaging studies published between 2014 and 2025 identified the caudate nucleus/lentiform nucleus as a core neural substrate of explicit outcome expectation effects, with the red nucleus emerging as an additional significant cluster.

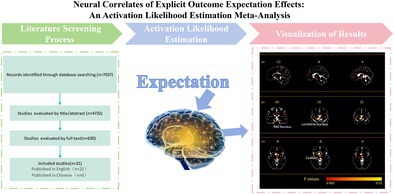

## 1 Introduction

Expectations constitute a central component of placebo effects in therapeutic contexts (Peerdeman et al. 2016; Colloca 2018). Treatment expectation effects are commonly defined as patients' future‐oriented cognitive representations of therapy‐related events or experiences (Laferton et al. 2017; Constantino et al. 2012; Li et al. 2024), encompassing expectations regarding treatment outcomes, treatment processes, and their perceived roles within the therapeutic context (Shedden‐Mora et al. 2023; Bingel et al. 2011; Hirsing et al. 2025). In experimental neuroimaging research, these phenomena are typically investigated using paradigms that manipulate explicit outcome expectations—consciously accessible predictions about forthcoming events or symptom relief, often induced via verbal instructions or contextual cues (Martin‐Pichora et al. 2011). While such paradigms capture a core operationalizable component of treatment expectation, they primarily emphasize its explicit, verbally mediated dimensions. Consequently, the neuroimaging literature to date most directly reflects the neural correlates of explicit outcome expectation effects. In clinical settings, placebo responses and treatment outcomes are substantially modulated by patients' expectations (Barth et al. 2019). Accumulating evidence indicates that positive expectations of analgesic efficacy can reduce opioid requirements in pain management (Bedford et al. 2021). Furthermore, significant associations have been reported between pretreatment expectations and clinical outcomes in postoperative functional recovery (Carriere et al. 2022) and inflammatory dermatoses (Sondermann et al. 2021). However, current research has predominantly focused on quantifying behavioral‐level effects through psychometric assessments and clinical observation, whereas the neurobiological correlates underlying explicit outcome expectation effects remain insufficiently understood.

The widespread application of functional neuroimaging techniques, including positron emission tomography (PET) and functional magnetic resonance imaging (fMRI), has enabled in vivo assessment of neuronal and neurochemical activity in the human brain (Ko et al. 2013; Atenas et al. 2018; De Lange et al. 2018). The flexibility of experimental paradigms has further contributed to their extensive use in cognitive neuroscience research (Liu 2012). In recent years, an increasing number of studies have employed these techniques to explore the neural correlates underlying expectation effects in therapeutic contexts. However, substantial heterogeneity in experimental paradigms has led to inconsistent findings regarding the patterns of brain activation. For instance, Kong et al. (2018), using an expectation manipulation paradigm, reported that enhanced expectations significantly enhanced functional connectivity between the nucleus accumbens (NAc) and medial prefrontal cortex (mPFC), the rostral anterior cingulate cortex (rACC), and dorsolateral prefrontal cortex (DLPFC). Similarly, Craggs et al. (2014) found that the expectation effects were associated with increased blood oxygen level–dependent (BOLD) activation in the lentiform nucleus, parahippocampal gyrus, and superior temporal gyrus during linguistically induced expectation.

To reconcile such discrepancies, coordinate‐based meta‐analytic approaches like activation likelihood estimation (ALE) have emerged as robust frameworks. This method models activation foci from independent neuroimaging studies as three‐dimensional Gaussian probability distributions, enabling probabilistic spatial convergence analysis through iterative kernel‐based smoothing and statistical thresholding (Acar et al. 2018). ALE is widely regarded as one of the most rigorous and commonly used coordinate‐based meta‐analytic methods (Yeung et al. 2023; Samartsidis et al. 2020). For example, Amanzio et al. (2013) identified consistent engagement of the anterior cingulate cortex (ACC) and periaqueductal gray (PAG) during anticipatory analgesia using ALE meta‐analyses. Notably, these findings suggest that expectation‐driven neural responses may reflect domain‐general neural mechanisms extending correlates beyond pain modulation.

Although expectation effects in pain regulation have been extensively explored, existing findings may primarily explain correlates specific to the pain domain, while their role in broader therapeutic behaviors remains unclear. Systematic investigation of domain‐general neural correlates underlying expectation effects is still lacking. Therefore, the present study conducted a coordinate‐based meta‐analysis of previous neuroimaging studies and applied dynamic FWHM adjustment and permutation testing in Ginger ALE 3.0.2 to identify the core neural correlates of explicit outcome expectation effects.

## 2 Materials and Methods

### 2.1 Literature Search and Exclusion Criteria

We systematically searched PubMed, Embase, Scopus, and Web of Science for studies published between January 1, 2014, and December 31, 2025. The 2014 cutoff was employed to enhance consistency in neuroimaging data acquisition, statistical correction, analytical standards (Atlas and Wager 2014; Haufe et al. 2013; Roiser et al. 2016), and conceptual definitions of expectation (Constantino et al. 2012). The following search terms were used in combination across all databases: ((fMRI[Title/Abstract]) OR (PET[Title/Abstract]) OR (brain activations[Title/Abstract]) OR (neuroimaging[Title/Abstract])) AND ((expect[Title/Abstract]) OR (expectation[Title/Abstract]) OR (placebo[Title/Abstract]) OR (anticipate[Title/Abstract])). Filters for clinical trials and randomized controlled trials were applied where available. Only studies published in English or Chinese were included. Additionally, the reference lists of all included articles were manually screened to identify any additional relevant studies.

The inclusion criteria were as follows:

1. Treatment expectation effects were defined as an individual's expectations or prediction regarding the effects of a specific treatment, intervention, or sensory stimulation in experimental or clinical contexts, encompassing both positive and negative expectancies. Accordingly, eligible studies included RCTs or controlled clinical trials investigating placebo effects or treatment expectations, in either clinical populations or healthy participants;
2. Participants' expectations, operationalized as explicit outcome expectations, were induced through verbal suggestion, conditioning, and guided imagery;
3. Studies using fMRI or PET that reported whole‐brain activation foci were included, whereas studies based solely on functional connectivity (FC) were excluded;
4. Results were reported in Talairach/Tournoux space or Montreal Neurological Institute (MNI) coordinates.

The exclusion criteria were as follows:

1. Non‐empirical studies (reviews, surveys, or case reports);
2. Studies using interventions unrelated to treatment expectation or placebo effects, such as those investigating only general prediction or vague expectations without an explicit treatment, intervention, or stimulation context;
3. Studies did not include whole‐brain analysis or were restricted to a limited region of interest (ROI).

### 2.2 Data Extraction

Reference management and data organization were performed using Clarivate EndNote and Microsoft Excel 2021. The following basic data were extracted from each study: publication details (e.g., authors, year of publication) and demographic characteristics of participants (e.g., sample size, age, and sex). Moreover, the method of expectation induction, the neuroimaging modality and main results were recorded. For studies that reported multiple contrasts, each contrast was treated as a separate experiment during data extraction; accordingly, the numbers of positive and negative activations reported herein reflect these independent activation contrasts rather than individual studies. Data extraction was performed independently by two investigators, with disagreements resolved through consultation with a third investigator when necessary.

### 2.3 Bias Assessment

In accordance with the quality assessment recommendations of the Cochrane Collaboration, two reviewers independently evaluated the risk of bias in the 21 included studies using the Risk of Bias (ROB) 2.0 tool and the ROBIN‐I tool. The ROB 2.0 tool was applied to RCTs and assessed five domains: the randomization process, the deviations from intended interventions, the missing outcome data, the measurement of the outcome, and the selection of the reported result. Non‐randomized studies were assessed using the ROBINS‐I tool, which evaluates seven domains: confounding, selection of participants, classification of interventions, deviations from intended interventions, missing data bias, measurement of outcomes, and selection of the reported results.

### 2.4 Activation Likelihood Estimation

The ALE method was used to perform a coordinate‐based neuroimaging meta‐analysis to identify brain regions showing cross‐study convergent activation associated with explicit outcome expectations (Eickhoff et al. 2009). ALE analyses were conducted using GingerALE 3.0.2 (http://brainmap.org/ale/) following the procedures below. First, activation coordinates were extracted from the included studies. Next, coordinates reported in non‐MNI spaces (e.g., Talairach) were converted to MNI152 standard space using the built‐in transformation function in GingerALE to ensure a common reference space. Then the standardized coordinates were entered into GingerALE for ALE meta‐analysis, and the optimized statistical protocol recommended by Eickhoff et al. (2016) was applied—specifically, we used a random‐effects ALE model with non‐additive correction and sample‐size‐dependent FWHM, with a whole‐brain gray matter mask (MNI152) applied. Subsequently, statistical significance was controlled at the cluster level using family‐wise error (FWE) correction, with the cluster‐forming threshold of *p* < 0.001 and a cluster‐level significance threshold of *p* < 0.05. Cluster‐level significance was estimated via permutation testing with 1000 iterations (Eickhoff et al. 2011; Heard and Lee 2019; Zhu et al. 2022). The key parameters of the ALE analysis are summarized in Table S4. Finally, significant activation clusters identified by GingerALE were extracted, including spatial coordinates, ALE values, and cluster volumes. The resulting ALE maps were subsequently imported into Mango software (https://mangoviewer.com/) and overlaid onto the standard MNI152 anatomical template to generate three‐dimensional visualizations of activation clusters.

### 2.5 Domain Classification and Sensitivity Analysis

To address potential biases arising from paradigm heterogeneity and to evaluate the robustness of the whole‐sample activation likelihood estimation (ALE) results, all 21 included studies were categorized a priori into four functionally distinct domains based on experimental paradigms and research objectives: Somatosensory Stimulation: Placebo Analgesia (*n* = 8), Somatosensory Stimulation‐Nocebo Hyperalgesia (*n* = 6), Affective and Antidepressant Expectancy (*n* = 3), and General Cognitive/Clinical Expectancy (*n* = 4). Given the limited sample size of studies within each domain, formal quantitative subgroup ALE analyses—which require sufficient statistical power to reliably detect convergent activation clusters within each subgroup—were not feasible. Instead, a qualitative synthesis of activation foci (i.e., the significantly activated brain regions reported in each individual study) was conducted for each domain to systematically summarize, organize, and compare the distribution and functional characteristics of single‐study activation foci across domains. Additionally, a leave‐one‐study‐out sensitivity analysis was performed to assess the stability of the whole‐sample ALE clusters: one study was sequentially removed from the dataset, and the ALE analysis was repeated on the remaining 20 studies at each iteration to examine whether the whole‐sample clusters were driven by any single study.

### 2.6 Open Access and Declarations

The full meta‐analytic protocol is available on PROSPERO (https://www.crd.york.ac.uk/PROSPERO/, registration number: CRD420251074894), where it was published as Version 1.0 on 16 June 2025. The study was registered prior to the initiation of the systematic search. The minimal dataset supporting the findings of this study is publicly available on the Open Science Framework (OSF) at https://osf.io/eazkw/.

## 3 Results

### 3.1 Literature Screening Process

**FIGURE 1:**
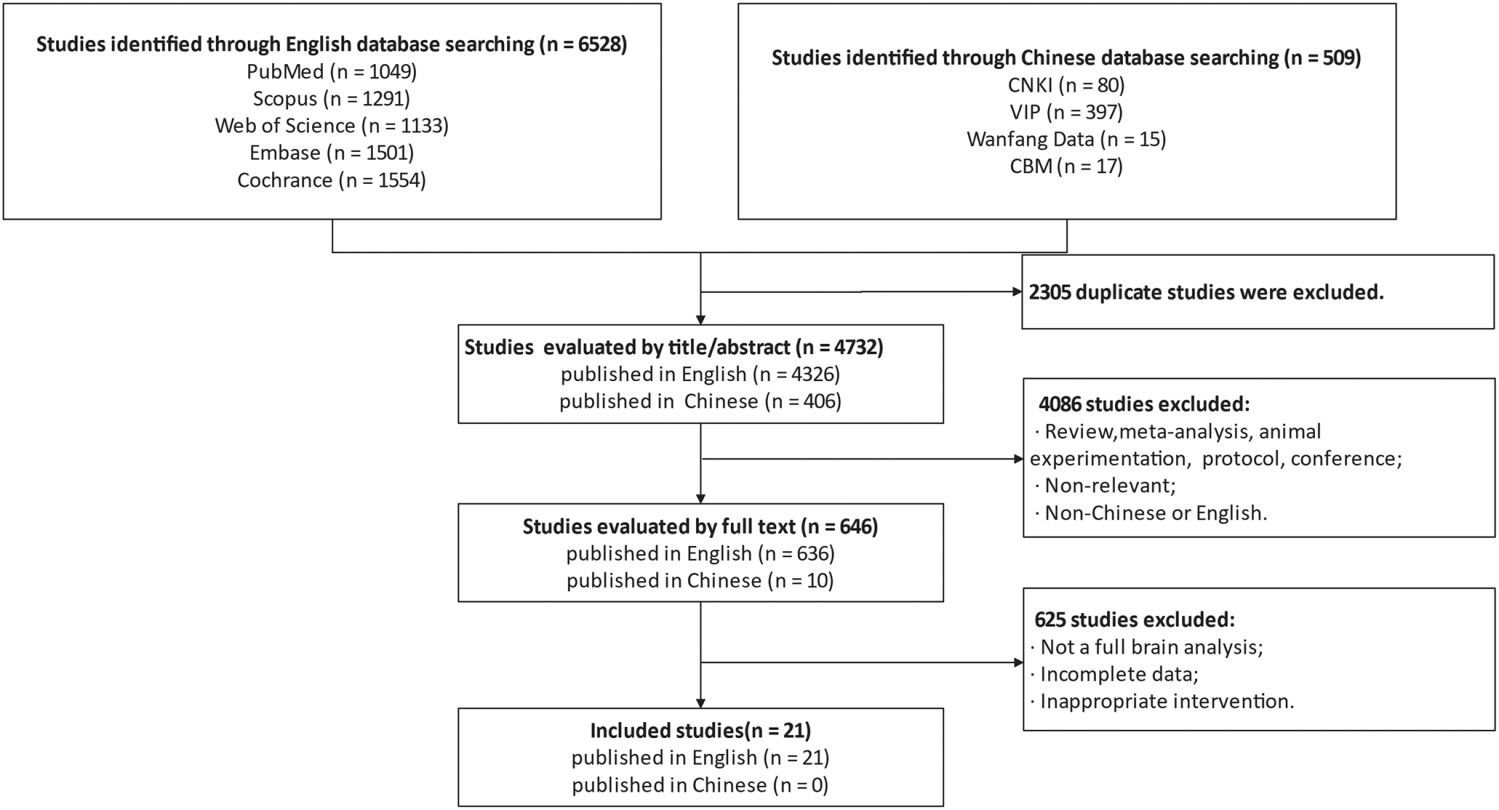
Flow diagram of the study selection.

A total of 7037 studies were identified through database searches. After removal of 2305 duplicates, 4086 studies were excluded during title and abstract screening. Following full‐text evaluation, 21 studies investigating explicit outcome expectations were included for further analysis. The literature selection process is summarized in Figure 1.

### 3.2 Procedure of Studies Evaluation

**FIGURE 2:**
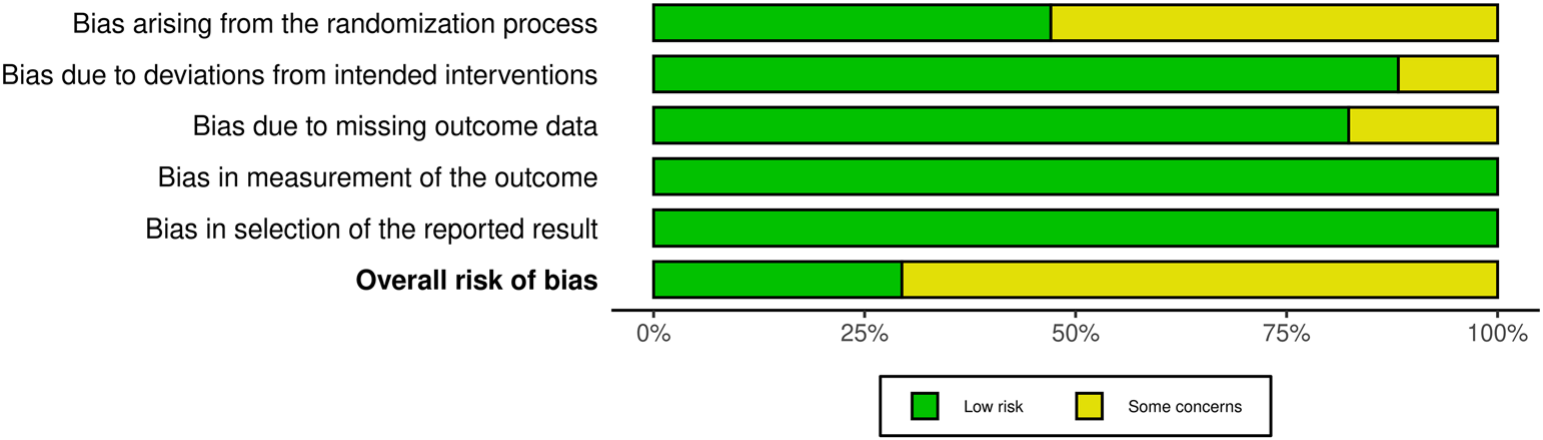
The risk of bias for 17 RCTs.

**FIGURE 3:**
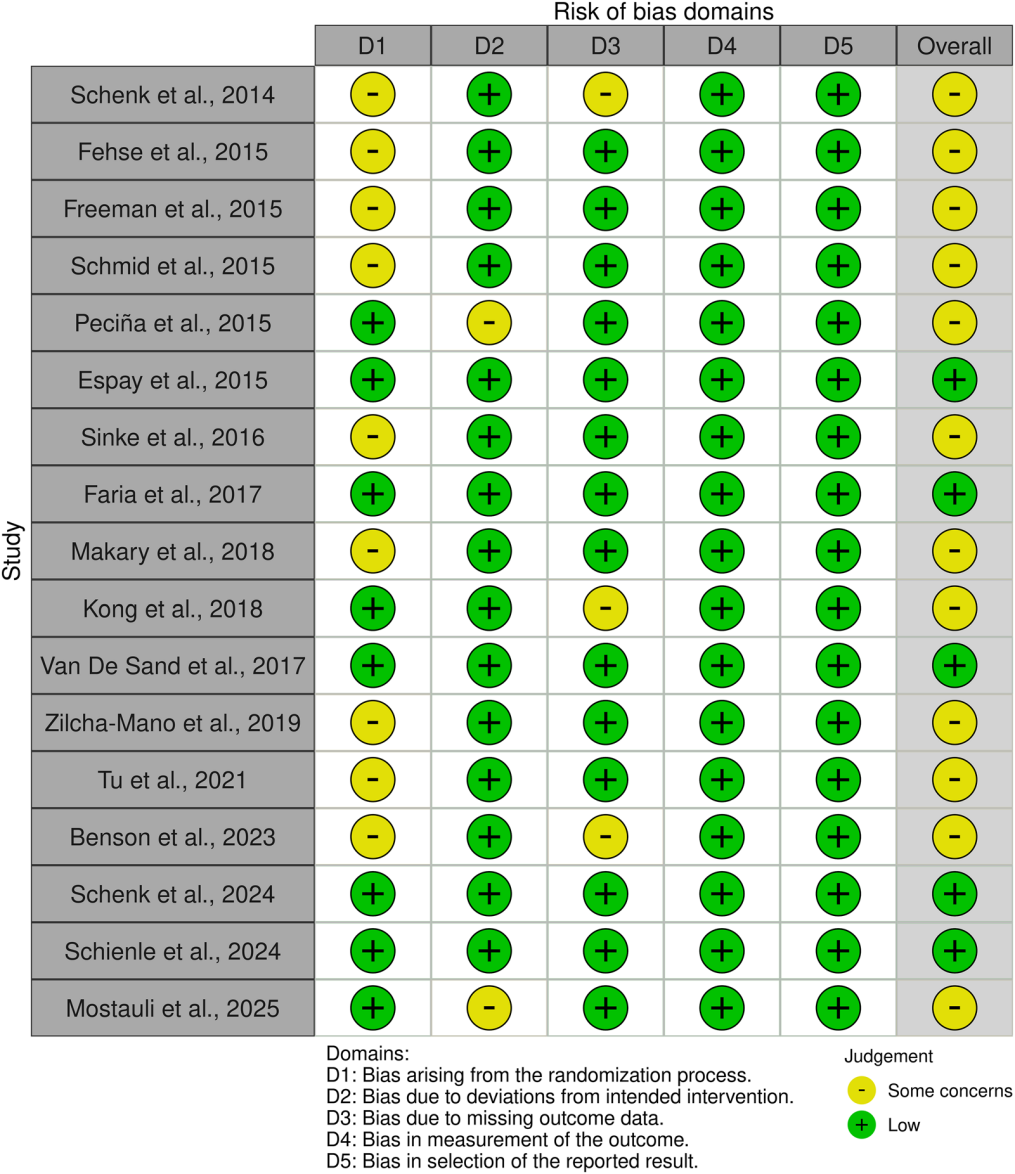
The risk of bias summary for 17 RCTs.

**FIGURE 4:**
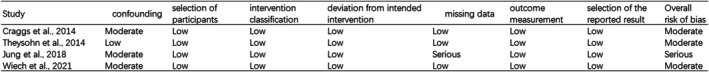
The risk of bias for four non‐randomized studies.

The ROB 2.0 tool was used to evaluate 17 RCTs, with detailed results presented in Figures 2 and 3. Overall, most studies demonstrated sound methodological quality; however, some concerns were noted regarding the randomization process, allocation concealment, blinding, and dropout rates. Five studies were judged to have a low overall risk of bias, whereas the remaining 12 studies raised some concerns. The ROBINS‐I tool was used to evaluate four non‐randomized studies, with detailed results shown in Figure 4. Of these, three were judged to have a moderate overall risk of bias and one a serious overall risk of bias. Collectively, approximately three‐quarters of the included studies were not at low risk of bias. Risk of bias visualizations were generated using the *robvis* tool (McGuinness and Higgins 2020).

### 3.3 The Basic Information of Studies Included

Among the 21 included studies, fMRI was used in 20 studies whereas PET was used in only one. Pain stimuli were incorporated into expectation manipulation in 10 studies, and the main neuroimaging findings involved the insula, frontal lobe, striatum, and other regions. Detailed information on the publication, demographic characteristics of the subjects, induction method of expectation, and main results are shown in Tables S1 and S2.

To further address issues related to the characteristics of expectation induction, assessment, and the corresponding neuroimaging timing windows, Table S3 systematically summarizes, for each included study, the targeted expectation dimension(s), specific expectation manipulation methods, psychometric tools for expectation assessment and their corresponding timing, details of expectation manipulation checks, outcome measures modulated by expectation, timing and measurement windows of outcome assessment, expected‐phase label, and ROI analysis result label. Notably, regarding the expected‐phase label, 20 studies were categorized as type A (anticipation under induced expectancy), type B (stimulus/outcome responses modulated by expectancy), or a combination of both. For expectation manipulation checks, some included studies did not perform formal assessments; specifically, one study (Faria et al. 2017) reported that expectancy assessments were not conducted continuously during treatment to avoid raising suspicion about the cover story. Table S3 clarifies methodological ambiguities related to expectation and provides a basis for evaluating the internal validity of included studies, supporting the rationale for data pooling across different paradigms.

### 3.4 The Results of ALE Meta‐Analyses

**FIGURE 5:**
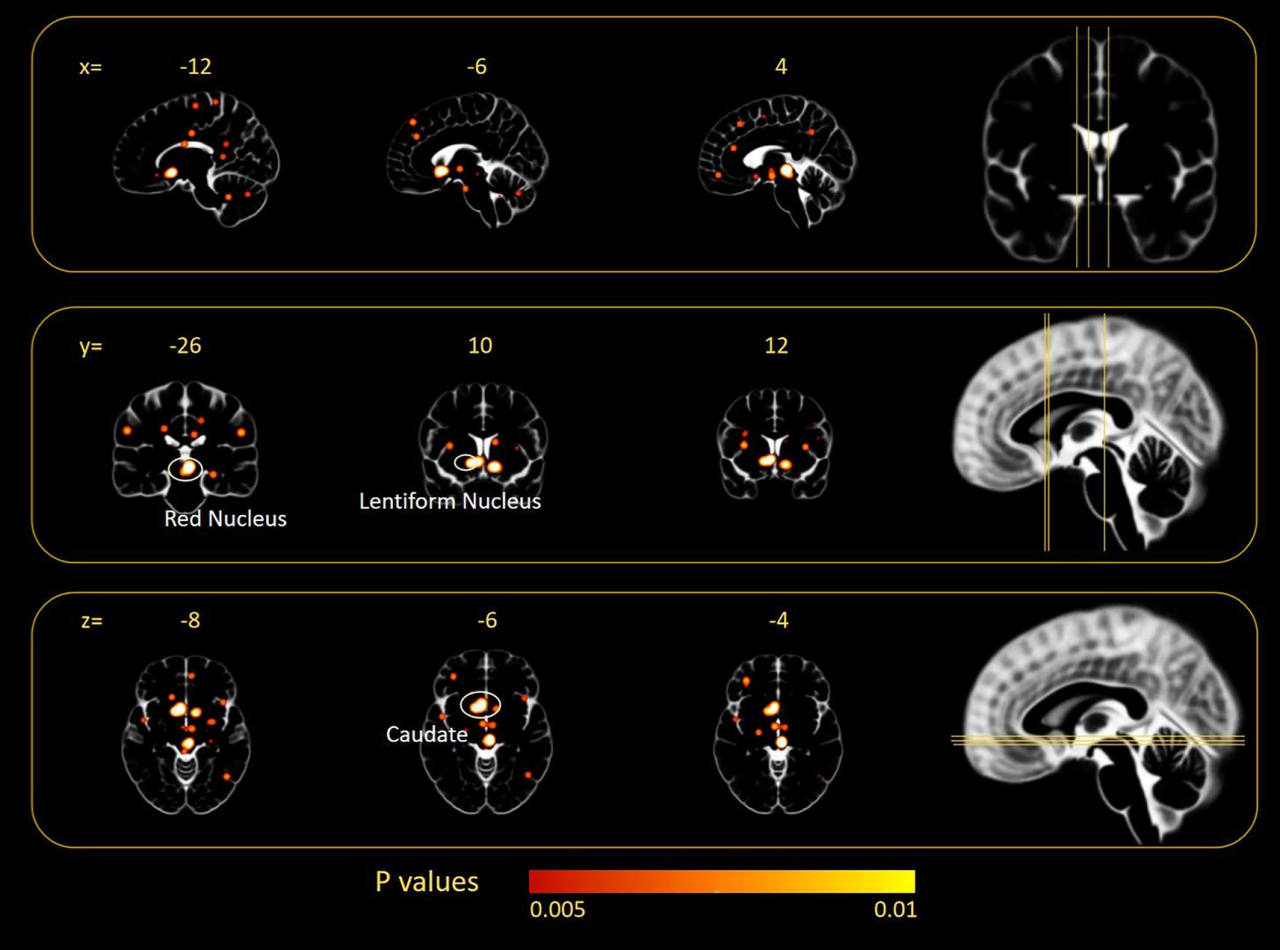
Areas of increased activity associated with the explicit outcome expectation.

A total of 21 studies were included in the ALE meta‐analysis, of which 18 reported positive activations and 5 reported negative activations. Among the positive activation analyses, two significant clusters of activation were identified (Cluster‐level FWE, *p* < 0.05):

Cluster 1 spanned the caudate nucleus and lentiform nucleus, and Cluster 2 was limitedly localized to the red nucleus, provided in Table 1 and Figure 5. No significant clusters were detected in the negative activation analyses. This lack of convergence may be explained by the limited number of studies reporting negative coordinates (*n* = 5), which reduced statistical power to detect spatially consistent effects.

**TABLE 1:** The detailed information for two significant clusters of activation.

Cluster	*x*	*y*	*z*	ALE	*P*	*Z*	Hemisphere	Label
1	−6	12	−6	0.0161	5.237725E‐6	4.4071264	L	Caudate
1	−12	10	−8	0.0154	9.275263E‐6	4.281664	L	Lentiform Nucleus
2	4	−26	−4	0.0230	1.9056772E‐8	5.499425	R	Red Nucleus

To further clarify the domain‐specific topography of brain activation and reduce biases from heterogeneity across paradigms, we performed a qualitative synthesis of activation foci across the four domains.

In the Somatosensory Stimulation—Placebo Analgesia domain (*n* = 8), consistent activation foci were observed in the anterior insula, anterior cingulate cortex (ACC), dorsolateral prefrontal cortex (DLPFC), secondary somatosensory cortex, and striatum. In the Somatosensory Stimulation—Nocebo Hyperalgesia domain (*n* = 6), convergent activation was observed in the anterior and posterior insula, ACC/midcingulate cortex (MCC), DLPFC/ventrolateral prefrontal cortex (VLPFC), amygdala, striatum, and brainstem nuclei, including the periaqueductal gray (PAG) and midbrain.

For the Affective and Antidepressant Expectancy domain (*n* = 3), activation foci were primarily localized within limbic and reward‐processing circuits, encompassing the amygdala, nucleus accumbens/subgenual ACC (NAc/sgACC), thalamus, and posterior cingulate gyrus. In the General Cognitive/Clinical Expectancy domain (*n* = 4), activation foci were distributed across multiple cortical regions, including the VLPFC, middle frontal gyrus, temporoparietal cortices, and pallidum, a component of the lentiform nucleus.

Notably, striatal activation was observed across all four domains, consistent with the core convergent cluster identified in the whole‐sample activation likelihood estimation (ALE) analysis.

### 3.5 Sensitivity Analysis

To assess the robustness of the whole‐sample ALE results, a leave‐one‐study‐out sensitivity analysis was conducted. In each iteration, one study was excluded, and ALE analyses were repeated on the remaining 20 studies. The convergent activation in the striatum and red nucleus remained stable across most iterations, supporting the robustness of the observed convergence patterns.

Additionally, a sensitivity analysis excluding studies with high risk of bias was performed. The main findings remained largely consistent with those of the whole‐sample analysis. The detailed results are provided in Table S5.

A further modality‐based sensitivity analysis was performed by excluding the single PET study. Following this exclusion, Cluster 1 was no longer significant, whereas the red nucleus remained stable in significance, peak coordinates, and cluster size (Table S6). This pattern may reflect differences in sensitivity and signal properties between fMRI and PET, particularly in the striatum, where PET may be more sensitive to dopaminergic and expectation‐related processes. In contrast, the red nucleus exhibited high stability across imaging modalities.

## 4 Conclusions

Through rigorous systematic searches, this study identified two significant activation clusters associated with the explicit outcome expectation effects: the caudate nucleus/lentiform nucleus and the red nucleus. These findings provide important insights into the neural correlates underlying explicit outcome expectation effects.

Both the caudate nucleus and lentiform nucleus are key components of the basal ganglia (Fazl and Fleisher 2018), which are extensively involved in behavior selection (Yin 2017), value processing (Kim and Hikosaka 2015), and reward anticipation (Hikosaka et al. 2006). The head of the caudate nucleus plays a central role in cognitive and emotional networks (Alexander et al. 1986) and participates in higher‐order cognitive processing via connections with the prefrontal cortex (Robinson et al. 2011; Pessoa 2008). In the context of explicit outcome expectations, the caudate nucleus may contribute to the cognitive processing of treatment‐related information and the formation of expectations through network connections with the frontal cortex. In addition, the caudate nucleus is closely linked to the reward system (Kawagoe et al. 1998; Hikosaka et al. 2014). Its activation observed in this study is consistent with hemodynamic evidence from Delgado et al. (2000), demonstrating distinct caudate responses to rewards and punishments. These findings suggest that the caudate nucleus, participating in the formation of expectations, may integrate treatment‐related information through the reward system, which is consistent with the present results. The lentiform nucleus, composed of the putamen and globus pallidus (De Barros et al. 2021), is involved in cognitive processes (Liuzzi et al. 2020) and reward processing (Wang et al. 2019). In this study, its functional characteristics were associated with the brain network activation patterns related to explicit outcome expectations, further supporting the role of the basal ganglia in the regulation of expectations.

In terms of explicit outcome expectation effects, the stable activation of the caudate nucleus and lentiform nucleus is not isolated. These structures are interconnected via gray matter extensions (Wilkinson 1992; Çırak et al. 2020), and their co‐activation may be closely related to the dynamic regulation of reward expectations (Haruno and Kawato 2006a). Using a heterarchical reinforcement learning model, Haruno and Kawato (2006b) further demonstrated that the caudate nucleus is primarily involved in learning processes based on comparing actual and predicted rewards, whereas the putamen primarily acquires stimulus‐action‐dependent reward predictions and may contribute to integrating reward expectation information and mediating reward‐oriented behaviors.

Because explicit outcome expectations are often accompanied by positive anticipation of treatment effects, such anticipation can be interpreted by the brain as a potential reward. This process engages the reward circuitry involving the caudate nucleus and may ultimately influence an individual's treatment motivation and adherence. The red nucleus, a midbrain structure (Massion 1988), is implicated in motor control (Basile et al. 2021) and cognitive processes (Nioche et al. 2009). A precise functional mapping study based on fMRI (Krimmel et al. 2025) proposed that the red nucleus may guide behavioral decisions based on motivational rewards or expectancy signals. However, this region has rarely been reported in previous meta‐analyses of expectation or placebo effects. Given that brainstem structures are particularly susceptible to spatial normalization and smoothing artifacts in coordinate‐based meta‐analyses, this convergence should be interpreted cautiously. Consequently, care must be taken to avoid false positives arising from methodological factors, and further clinical studies are warranted to validate the specific functional role of the red nucleus.

To clarify domain‐specific characteristics of expectation effects, qualitative synthesis of activation foci was conducted across four domains. “Somatosensory Stimulation: Placebo Analgesia” was characterized by activation in cortical pain‐modulatory regions and the striatum, consistent with its role in sensory expectation and pain relief. “Somatosensory Stimulation: Nocebo Hyperalgesia” involved pain‐modulatory, limbic, and brainstem regions alongside the striatum, reflecting the aversive nature of sensory expectation processes. “Affective and Antidepressant Expectancy” was primarily associated with limbic and reward circuitry, with striatal activation supporting emotional and antidepressant‐related expectancy. “General Cognitive/Clinical Expectancy” was accompanied by striatal involvement in cognitive and non‐sensory expectancy. Notably, the striatum was consistently activated across all four domains, indicating that it serves as a shared core substrate for expectation effects. This universality highlights the striatum's central role in mediating common psychological processes underlying these diverse expectations, such as expectation encoding and reward prediction, whereas domain‐specific activation patterns reflect functional specialization that adapts to sensory, affective, or cognitive demands.

Notably, relatively prominent activations in the frontal lobe and insular regions were also observed in the visualization images. These regions are implicated in comparing psychological expectations with actual experiences and jointly contribute to reward expectation (Knutson and Greer 2008; Sherman et al. 2016; Osada et al. 2024). However, the activation levels in these regions did not reach statistical significance, which may be attributable to the limited number of included studies. The present findings diverge from prior meta‐analyses of placebo analgesia and expectation, which consistently reported activation in the anterior cingulate cortex (ACC), insula, periaqueductal gray (PAG), and prefrontal regions (Atlas and Wager 2014; Zunhammer et al. 2021). Notably, the current ALE results highlight the striatum (caudate/lentiform nucleus) as a core node, with an additional cluster observed in the red nucleus, without significant activation in the ACC, insula, or PAG. This divergence likely reflects the distinct focus of the present study: whereas previous meta‐analyses emphasized pain‐specific placebo/analgesia effects which rely heavily on somatosensory and nociceptive regulation mediated by the ACC, insula, and PAG, the current study targets general explicit outcome expectation effects across diverse clinical domains. The unique emphasis on the striatum and red nucleus may therefore reflect a shift toward reward, motivational, and motor preparatory processing—key components of general explicit outcome expectation—rather than the somatosensory and nociceptive circuitry prioritized in pain‐specific placebo research. The absence of significant convergence in these canonical regions may also stem from several methodological factors, including limited statistical power, coordinate dispersion due to paradigm heterogeneity, and differences in inclusion criteria.

Several limitations should be acknowledged. First, although all included studies pertain to explicit outcome expectation effects, substantial heterogeneity existed in participants, interventions, and measurement methods. This heterogeneity, combined with the limited number of studies within each domain, precluded formal subgroup ALE analyses and may have reduced the anatomical specificity of the findings. Relatedly, the inclusion of both clinical patients and healthy volunteers, along with the relatively small sample sizes (*n* < 20 per group), constrains the generalizability of the results across different populations. Second, the analysis relied primarily on whole‐brain activation coordinates, potentially overlooking functional connectivity between regions. Third, publication bias cannot be excluded, as some studies did not report negative activation coordinates, which may bias the results toward positive activations. Finally, approximately three‐quarters of included studies were not at low risk of bias according to the ROB2.0 and ROBINS‐I assessments. Potential biases from inadequate randomization, blinding, or selective reporting may inflate convergent activation patterns and reduce confidence in the findings. Nevertheless, sensitivity analyses indicated that the majority of core results remained stable, supporting the robustness of the main activation clusters. Taken together, these limitations underscore the need for future high‐quality, large‐sample studies to generate more reliable and generalizable conclusions in this field. Despite these limitations, the present findings provide novel insights into the neural correlates underlying explicit outcome expectation effects and advance our understanding of how the brain processes this information. Qualitative synthesis across four domains further revealed that the striatum, a key structure of the basal ganglia, was consistently activated across all domains, supporting its role as a shared neural substrate for explicit outcome expectation effects. Distinct domain‐specific activation patterns were also observed, reflecting functional specialization for different expectancy contexts. Importantly, elucidating the contributions of these brain regions may inform the development of interventions aimed at enhancing expectation effects and improving clinical outcomes. Future studies should employ larger sample sizes to investigate the functional connectivity between relevant brain regions more comprehensively. In addition, integrating behavioral and physiological measures will be critical to fully characterize the correlates and impact of expectation effects.

## Funding

This work was supported by the National Natural Science Foundation of China (81904063).

## Conflicts of Interest

The authors declare no conflicts of interest. All authors have reviewed and approved the final version of the manuscript.

## Supporting information


**Table S1:** Overview of the included studies on publication details and demographic characteristics of the subjects.
**Table S2:** Overview of the included studies on induction method of expectation and main results.
**Table S3:** Overview of the included studies on expectation‐related characteristics, assessment timing and manipulation check.
**Table S4:** Key parameters of GingerALE 3.0.2 for ALE meta‐analysis.
**Table S5:** The detailed information from sensitivity analysis excluding studies with high risk of bias.
**Table S6:** The detailed information from sensitivity analysis excluding the PET study.

## Data Availability

Partial data supporting the findings of this study are available in the Supporting Information. The remaining data are available from the corresponding author upon reasonable request.
