# Supplementary material for: Neural Correlates of Explicit Outcome Expectation Effects: An Activation Likelihood Estimation Meta‐Analysis
Source: Hum Brain Mapp. 2026 Jul 2;47(10):e70594. doi: 10.1002/hbm.70594 (PMC13328837; doi:10.1002/hbm.70594)
Supplement: Supplementary file 1 — Table S1: Overview of the included studies on publication details and demographic characteristics of the subjects. Table S2: Overview of the included studies on induction method of expectation and main results. Table S3: Overview of the included studies on expectation‐related characteristics, assessment timing and manipulation check. Table S4: Key parameters of GingerALE 3.0.2 for ALE meta‐analysis. Table S5: The detailed information from sensitivity analysis excluding studies with high risk of bias. Table S6: The detailed information from sensitivity analysis excluding the PET study. [file HBM-47-e70594-s001.doc]

**Supplementary Table S1.**

*Overview of the Included Studies on Publication Details and Demographic Characteristics of the Subjects*

| study | country | Subjects | | |
| --- | --- | --- | --- | --- |
|  |  | n | mean age | sex |
| Craggs et al., 2014 | USA | Enhance placebo=9 Standard placebo=15 | Enhance placebo=27.7±9.6 Standard placebo=31.6±8.6 | Enhance placebo=9F Standard placebo=15F |
| Schenk et al., 2014 | Germany | open treatment=hidden treatment=placebo=control=32 | 25.6±3.3 | 15F |
| Theysohn et al., 2014 | Germany | Women=15 Men=15 | Women=36.9±3.6 Men=32.4±2.8 | Women=15F Men=0F |
| Fehse et al., 2015 | Germany | 30 | 32±6.39 | 0F |
| Freeman et al., 2015 | USA | 24 | 21~49 | 12F |
| Schmid et al., 2015 | Germany | NOCEBO=28 COUNTROL=16 | NOCEBO=25.29 ± 4.47 COUNTROL=24.00 ± 1.67 | NOCEBO=14F COUNTROL=8F |
| Peciña et al., 2015 | USA | Active Placebo=Inactive Placebo=35 | 35±13 | 23F |
| Espay et al., 2015 | USA | “cheap” placebo= “expensive” placebo=12 | 62.4±7.9 | 3F |
| Sinke et al., 2016 | Germany | positive expectancy group=22 negative expectancy group=21 | 25.25 | 23F |
| Faria et al., 2017 | Sweden | Overt SSRI=24 covert SSRI=22 | Overt SSRI=31.0±10.6 covert SSRI=32.0±9.7 | Overt SSRI=9F covert SSRI=9F |
| Jung et al., 2018 | Republic of Korea | 22 | 23.7±3.3 | 8F |
| Makary et al., 2018 | Republic of Korea | REAL=28 PHNT=19 | REAL=38.7±13.1 PHNT=39.5±13.7 | REAL=12F PHNT=10F |
| Kong et al., 2018 | USA | Boosted Acu=17 Standard Acu=17 TAU=12 | Boosted Acu=61.3 ± 6.9 Standard Acu=61.2 ± 7.7 TAU=60.1 ± 7.1 | Boosted Acu=9F Standard Acu=10F TAU=8F |
| Van De Sand et al., 2017 | Germany | Itch-nocebo= itch-only=30 | 25.5 | 18F |
| Zilcha-Mano et al., 2019 | USA | Open group=9 Placebo-controlled group=14 | 40.04 ± 8.29 | 13F |
| Tu et al., 2021 | USA | Anodal tDCS=27 Cathodal tDCS=27 Sham tDCS=27 | Anodal tDCS=27.4±6.3 Cathodal tDCS=26.9±5.9 Sham tDCS=27.9±7.1 | Anodal tDCS=13F Cathodal tDCS=11F Sham tDCS=13F |
| Wiech et al., 2021 | UK | 22 | 25.95±4.20 | 11F |
| Benson et al., 2023 | Germany | LPS= Placebo=39 | 26.0±0.7 | 17F |
| Schenk et al., 2024 | Germany | Expectancy group=42 No-Expectancy group=35 | Expectancy group=24.3±3.8  No-Expectancy group=24.8±5.4 | Expectancy group=30F  No-Expectancy group=21F |
| Schienle et al., 2024 | Austria | OLP pill=33 Imaginary pill=33 PV=33 | Imaginary Pill=21.45± 3.08 OLP Pill=23.85±6.97 PV=24.15±7.39 | 99F |
| Mostauli et al., 2025 | Germany | Placebo group= Control group=49 | 26.92±4.23 | 30F |

**Supplementary Table S2.**

*Overview of the Included Studies on Induction Method of Expectation and Main Results*

| study | Intervention | neuroimaging method | Main results |
| --- | --- | --- | --- |
|
| Craggs et al., 2014 | painful rectal distensions+medicine+verbal instructions | fmri | Compared to s-PL, e-PL showed significantly increased BOLD activation in the lentiform nucleus, parahippocampal gyrus, and superior temporal gyri. |
| Schenk et al., 2014 | heat pain stimuli+medicine+treatment expectancy manipulations | fmri | Interaction between treatment and expectation was observed in the anterior insular cortex, subgenual anterior cingulate cortex, and ventral striatum. |
| Theysohn et al., 2014 | painful rectal distensions+placebo+verbal instructions | fmri | 100% expectation condition led to increased activation of VLPFC, anterior insula, DLPFC, and superior parietal gyrus, and decreased insula activation during pain anticipation. |
| Fehse et al., 2015 | placebo+verbal suggestions | fmri | Direct comparison of placebo conditions showed higher activation in bilateral dorsolateral (and dorsomedial) prefrontal cortex in the original brand group. |
| Freeman et al., 2015 | pain stimuli+inert creams | fmri | Expectation of pain relief evoked significant fMRI signal changes in the striatum. |
| Schmid et al., 2015 | pain stimuli+verbal instructions | fmri | In the nocebo group, increased expected and perceived pain intensity was paralleled by enhanced activation of the secondary somatosensory cortex and amygdala during cued pain anticipation. |
| Peciña et al., 2015 | placebo+open-label trial | PET | Significant differences in the active placebo group were found in the subgenual anterior cingulate cortex, nucleus accumbens, midline thalamus, and amygdala. |
| Espay et al., 2015 | placebo+verbal instructions | fmri | Expensive placebo decreased brain activation; cheap placebo increased activation in bilateral anterior/posterior cingulate cortices, left lateral sensorimotor cortex, right parietal cortex and other regions. |
| Sinke et al., 2016 | Heat pain stimuli+verbal and written instructions | fmri | Increased short-term memory in the positive expectancy group was associated with activity in the right SPC/IPC. |
| Faria et al., 2017 | medicine+verbal instructions | fmri | Overt SSRI arm showed increased reactivity in bilateral posterior cingulate cortex, left mid temporal gyrus, and left inferior frontal gyrus. |
| Jung et al., 2018 | pseudo-electrical stimulation+visual information | fmri | Expectations of acupuncture stimulation were associated with insula and pre-supplementary motor area activation. |
| Makary et al., 2018 | either real (REAL) or phantom (PHNT) acupuncture stimulation | fmri | In the PHNT group, belief in acupuncture effectiveness positively correlated with right dlPFC and vlPFC activation. |
| Kong et al., 2018 | acupuncture+expectancy manipulation model | fmri | Boosted group showed differences in medial prefrontal cortex (MPFC)/rostral anterior cingulate cortex (rACC) and dorsolateral prefrontal cortex compared to the standard acupuncture group. |
| Van De Sand et al., 2017 | Itch paradigm+verbal instruction | fmri | Nocebo vs. control showed increased activity in the contralateral (right) rolandic operculum. |
| Zilcha-Mano et al., 2019 | medicine+verbal instructions | fmri | Significant inter-condition differences were found in neural activation of the amygdala, superior temporal gyrus, insula, and thalamus. |
| Tu et al., 2021 | tDCS+expectancy manipulation model | fmri | Placebo contrast showed increased activation in the caudate, pre-SMA, ACC, vmPFC, and rDLPFC, and decreased activation in the SMA and S1. |
| Wiech et al., 2021 | noxious stimulation+visual cues | fmri | Dorsolateral prefrontal cortex, amygdala, and periaqueductal gray are involved in these processes. |
| Benson et al., 2023 | visceral pain stimuli+medicine+predictive visual conditioning cues | fmri | During cued pain anticipation, inflammation × mood interaction affected activation of bilateral caudate nucleus and right hippocampus. |
| Schenk et al., 2024 | painful heat stimuli+placebo | fmri | In the Expectancy group, coupling between rACC and PAG was increased during active vs. inert placebo compared to the No-Expectancy group. |
| Schienle et al., 2024 | open-label placebos | fmri | Imaginary Pill group showed lower activity in the insula, pallidum, and VLPFC than the PV group. |
| Mostauli et al., 2025 | placebo+verbal instruction | fmri | Expectancy-related placebo is mediated by prefrontal-limbic networks |

**Supplementary Table S3.**

*Overview of the Included Studies on Expectation-Related Characteristics, Assessment Timing and Manipulation Check*

| study | Expectation Dimension | Expectation Manipulation | Expectation Assessment Tool | Timing of Expectation Assessment | Expectation Manipulation Check | Outcome Measure Modulated by Expectation | Timing of Outcome Assessment | Measurement Window | Expected Phase Label | ROI Analysis Result Label |
| --- | --- | --- | --- | --- | --- | --- | --- | --- | --- | --- |
| Craggs et al., 2014 | outcome expectation | pain stimuli+verbal suggestion+placebo | / | / | / | individual pain ratings | within 10s after termination of the scan and last intervention. | during stimulus presentatio | b | Enhanced Placebo vs Standard Placebo |
| Schenk et al., 2014 | outcome expectation | pain stimuli+placebo | Questionnaire(anticipated pain expectancy with/without treatment) | before the intervention | the expectancy conditions vs the no-expectancy conditions, F(1,31)=20.5，p < 0.05 | VAS(pain intensity) | before the intervention | pre-pain stimulus anticipation period + during pain stimulus presentation | a+b | (open treatment<hidden treatment) vs (placebo<control) |
| Theysohn et al., 2014 | outcome expectation | pain stimuli+verbal instructions | VAS (expected pain, present tension) | prior to each session | the 0% expectation condition vs the 100% expectation condition, F=5.15，p < 0.05 | VAS(pain intensity) | after each session | during cued anticipation and pain delivery | a+b | the 0% expectation condition vs the 100% expectation condition |
| Fehse et al., 2015 | outcome expectation | heat pain+verbal instructions | / | / | / | NRS(pain intensity) | after each of the two runs | After placebo | b | original vs generic analgesic |
| Freeman et al., 2015 | outcome expectation | pain stimuli | Subjective Rating (intervention-related pain relief/enhancement expectancy) | before/after the intervention, and after the scan was complete. | Positive Expectancy vs Negative Expectancy, F (4,92) = 26.60, p < .001 | the Gracely Scales(pain intensity) | before/after the intervention | during cued anticipation and pain delivery | a+b | In the expectation condition, pre vs post |
| Schmid et al., 2015 | outcome expectation | pain stimuli+verbal instructions | VAS(tension and expected pain) | Prior to each session | the nocebo group vs the control group, F = 4.054, p = 0.050 | Likert-type scale/VAS Scales(pain intensity) | During scanning | during pain anticipation | a | the nocebo group vs the control grou |
| Peciña et al., 2015 | outcome expectation | verbal instructions+open-label placebo | / | / | / | QIDS-SR16 | pre- and post- each placebo intervention | After each placebo week | c | “active” placebo vs “inactive” placebo |
| Espay et al., 2015 | outcome expectation | verbal instructions+ placebo | / | / | / | UPDRS-III/Hoehn and Yahr staging /CGI and so on | At visit 1 and after the intervention | during task performance | b | expensive placebo vs cheap placebo |
| Sinke et al., 2016 | outcome expectation | verbal and written instructions | VAS(mental state); STAI/PVAQ/PASS-D/PCS | After the calibration stage | negative expectancy vs positive expectancy,t(41)= 6.4,p=0.001 | VAS(pain intensity) | after each stimulus | during cued anticipation and pain delivery | a+b | negative expectancy vs positive expectancy |
| Faria et al., 2017 | outcome expectation | Verbal Suggestions | / | / | / | LSAS-SR | Before and after the full treatment period | during task performance | b | Overt vs covert SSRI |
| Jung et al., 2018 | outcome expectation | visual information+pseudo-stimulation | / | / |  | VAS (De qi sensation) | after each stimulus | during stimulus anticipation | b | pseudo-stimulation vs the control condition |
| Makary et al., 2018 | outcome expectation | auditory cue+visual information | acupuncture efficacy expectation scores | Before the fMRI runs | / | MASS/VAS(pain intensity) | before and after the stimulation | during the acupuncture stimulation | b | PHNT vs REAL |
| Kong et al., 2018 | outcome expectation | expectancy manipulation model(pain stimuli+verbal suggestion) | VAS (expected relief of exogenous heat pain, endogenous chronic knee pain) | at baseline, before and after the stimulation, and at the end of the study | Boosted vs Standard vs TAU Acupuncture, F(2,42) = 7.04，p = 0.002 | KOOS | at baseline, the midpoint, and the end of the study | before/after the intervention | b | Boosted vs Standard vs TAU |
| Van De Sand et al., 2017 | outcome expectation | Verbal Suggestions+ temperature stimuli | / | / | / | VAS(itch score) | in the last of each temperature block | during testing sessions | b | Itch-nocebo vs itch-only |
| Zilcha-Mano et al., 2019 | outcome expectation | Verbal Suggestions | the Treatment Credibility and Expectancy Scale (CES) | before/after the intervention | Open vs. Placebo-controlled group, W=31.5, p=0.007 | HRSD | before/after the intervention | during task performance | b | Open vs. Placebo-controlled group |
| Tu et al., 2021 | outcome expectation | expectancy manipulation model (pain stimuli+images cues) | ERS(changes of expectation for pain decrease) | before/after the intervention | the anodal(1.70) vs cathodal(2.30) vs sham tDCS(1.67 ),p<0.01 | the Gracely Scales(pain intensity) | during the stimulation | during pain experience | b | Lidocaine vs Neutral |
| Wiech et al., 2021 | outcome expectation | visual cues+pain stimuli | / | / | / | / | / | during stimulus anticipatio+during the stimulation period | a+b | High pain bias group vs Low pain bias group |
| Benson et al., 2023 | outcome expectation | visual cues+pain stimuli | VAS(mood state) | baseline, prior and after scanning | mood condition X time interaction,F(2;74)= 41.0，p <0.001 | VAS(pain intensity) | after the intervention | During cued pain anticipation | a | pain anticipation-Interaction Inflammation x Mood |
| Schenk et al., 2024 | outcome expectation | Verbal Suggestions+ capsaicin or saline+thermal pain | Questionnaire (intervention expectation: believed no-treatment/no belief/believed treatment) | after the runs | the Expectancy vs the No-Expectancy group,P = 0.04;F（1,75） = 4.3 | VAS(pain intensity) | before/after the intervention | during testing sessions | b | the Expectancy vs the No-Expectancy group |
| Schienle et al., 2024 | outcome expectation | open-label placebos+Image intervention | Questionnaire (expectations concerning the effectiveness of the intervention, 1-9 scale) | before the intervention | Expected efficacy, OLP pill(5.39) vs Imaginary pill(6.48) | Questionnaire (the perceived effectiveness of the treatment, 1-9 scale) | at the end of the experiment | during testing sessions | b | OLP Pill vs Imaginary Pill vs Passive Viewing |
| Mostauli et al., 2025 | outcome expectation | placebo+verbal instruction | an 11-point-scale(Expectation of positive mood change) | after placebo application | placebo vs control,T(48) = 7.90, p < 0.001 | VAS(Mood state) | Before scanning, after each scanning block | during testing sessions | b | placebo vs control |

*Note.* Expected Phase Label: (a) anticipation under induced expectancy; (b) stimulus/outcome responses modulated by expectancy; (c) resting-state/FC changes after expectancy induction.

**Supplementary Table S4.**

*Key Parameters of GingerALE 3.0.2 for ALE Meta-Analysis*

| Parameter | Setting |
| --- | --- |
| Analysis mode | Single Dataset |
| Coordinate space | MNI152 standard space |
| ALE model | Random-effects ALE with non-additive correction |
| FWHM | Sample-size-dependent |
| Brain mask | Whole-brain gray matter mask (MNI152) |
| Threshold method | Cluster-level Inference |
| Cluster-forming threshold | Uncorrected p < 0.001 |
| Cluster-level FWE-corrected threshold | p < 0.05 |
| Thresholding Permutations | 1000 |
| Minimum cluster size | 568 mm³ |

**Supplementary Table S5.**

*The Detailed Information from Sensitivity Analysis Excluding Studies with High Risk of Bias*

| Cluster | x | y | z | ALE | P | Z | Hemisphere | Label |
| --- | --- | --- | --- | --- | --- | --- | --- | --- |
| 1 | -6 | 12 | -6 | 0.016086148 | 4.892542E-6 | 4.4218764 | L | Caudate |
| 1 | -12 | 10 | -8 | 0.015425869 | 8.693892E-6 | 4.2960434 | L | Lentiform Nucleus |
| 2 | 4 | -26 | -4 | 0.022953002 | 1.7291422E-8 | 5.5165434 | R | Red Nucleus |

**Supplementary Table S6.**

*The Detailed Information from Sensitivity Analysis Excluding the PET Study.*

| Cluster | x | y | z | ALE | P | Z | Hemisphere | Label |
| --- | --- | --- | --- | --- | --- | --- | --- | --- |
| 1 | 4 | -26 | -4 | 0.022952992 | 1.6407448E-8 | 5.525763 | R | Red Nucleus |

**List of Abbreviations**

PHNT = Phantom acupuncture

Acu = Acupuncture

TAU = Treatment As Usual

OLP = Open-Label Placebo

VLPFC = Ventrolateral Prefrontal Cortex

DLPFC = Dorsolateral Prefrontal Cortex

tDCS = transcranial Direct Current Stimulation

SPC = Superior Parietal Cortex

IPC = Inferior Parietal Cortex

MPFC = Medial Prefrontal Cortex

pre-SMA = pre-Supplementary Motor Area

ACC = Anterior Cingulate Cortex

vmPFC = ventromedial Prefrontal Cortex

SMA = Supplementary Motor Area

PAG = Periaqueductal Gray

VAS = Visual Analogue Scale

NRS = Numerical Rating Scale

QIDS-SR16 = Quick Inventory of Depressive Symptomatology-Self Rated 16

STAI = State-Trait Anxiety Inventory

PVAQ = Pain Vigilance and Awareness Questionnaire

PASS-D = Pain Anxiety Symptoms Scale-Daily

PCS = Pain Catastrophizing Scale

LSAS-SR = Liebowitz Social Anxiety Scale-Self Report

MASS = Measures of Acupuncture Sensations Scale

KOOS = Knee Injury and Osteoarthritis Score

HRSD = Hamilton Depression Scale

ERS = Expectations for Relief Scale

ROI = Region of Interest

ALE = Activation Likelihood Estimation
